# Green Energy Storage: Chitosan-Avocado Starch Hydrogels for a Novel Generation of Zinc Battery Electrolytes

**DOI:** 10.3390/polym15224398

**Published:** 2023-11-14

**Authors:** María I. Cruz-Balaz, María Fernanda Bósquez-Cáceres, Anabel D. Delgado, Noé Arjona, Vivian Morera Córdova, Lorena Álvarez-Contreras, Juan P. Tafur

**Affiliations:** 1 Grupo de Investigación Aplicada en Materiales y Procesos (GIAMP), School of Chemical Sciences & Engineering, Yachay Tech University, Urcuquí 100115, Ecuador; maria.cruzb@yachaytech.edu.ec (M.I.C.-B.); maria.bosquez@yachaytech.edu.ec (M.F.B.-C.); vmorera@yachaytech.edu.ec (V.M.C.); 2 Centro de Investigación en Materiales Avanzados S.C. (CIMAV), Miguel de Cervantes No. 120, Complejo Industrial Chihuahua, Chihuahua 31136, Mexico; anabel.delacruz@cimav.edu.mx; 3 Centro de Investigación y Desarrollo Tecnológico en Electroquímica S. C., Pedro Escobedo, Querétaro C.P. 76703, Mexico; wvelazquez@cideteq.mx; 4 Departamento de Ingeniería Mecánica, Química y Diseño Industrial, Escuela Técnica Superior de Ingeniería y Diseño Industrial (ETSIDI), Universidad Politécnica de Madrid (UPM), Ronda de Valencia 3, 28012 Madrid, Spain

**Keywords:** chitosan–starch hydrogels, zinc–air batteries, crosslinking methods, freezing strategies

## Abstract

Meeting the ever-increasing global energy demands through sustainable and environmentally friendly means is a paramount challenge. In response to this imperative, this study is dedicated to the development of biopolymer electrolytes, which hold promise for improving the efficiency, safety, and biodegradability of energy systems. The present study aims to evaluate hydrogels synthesized from chitosan biopolymer and starch from avocado seed residues in different ratios, and dried using freeze-thawing and freeze-drying techniques. Epichlorohydrin was used as a chemical crosslinker to create a suitable degree of swelling using an ionic solution. Physical freezing crosslinking strategies such as freezing–thawing and freezing–drying were performed to generate a denser porous structure in the polymer matrix. Subsequently, synthesized electrolytes were immersed in 12 M KOH solution to improve their electrochemical properties. The effect of the different ratios of starch in the hydrogels on the structural properties of the materials was evaluated using characterization techniques such as FTIR and XRD, which allowed to confirm the crosslinking between chitosan and starch. The electrochemical performance of the hydrogels is assessed using electrochemical impedance spectroscopy. A maximum conductivity value of 0.61 S·cm^−1^ was achieved at room temperature. The designed materials were tested in prototype zinc–air batteries; their specific capacity value was 1618 mA h·g^−1^, and their obtained power density was 90 mW·cm^−2^. These substantial findings unequivocally underscore the potential of the synthesized hydrogels as highly promising electrolytes for the application in zinc–air battery systems.

## 1. Introduction

The imperative need to meet the current energy demands with advanced energy storage solutions stems from the call for mitigating the negative impacts of fossil fuel-based energy systems. The finite availability of fossil fuels and their contribution to greenhouse gas emissions are some of the main issues to be addressed [1]. The primary solution that comes to mind is renewable energy sources. However, their intermittent nature, such as in solar and wind energies, underscores the importance of effective energy storage technology to ensure a steady and reliable energy supply [2]. Different electrochemical energy storage systems that can meet the requirements for the storage of renewable energy including sodium–sulfur batteries, lithium-ion batteries, cost-effective redox-flow batteries, and recently, novel zinc–air batteries (ZABs) have been proposed [3,4]. ZABs are appealing because of their environmental friendliness, low cost, and high energy density [5]. Their battery functions by harnessing the electrochemical reactions between zinc and oxygen in order to generate electrical energy, a promising choice for grid-scale energy storage [6].

The incorporation of polymeric electrolytes into metal–air batteries, including ZABs, is emerging as a more efficient solution for energy storage. To fully exploit the potential of ZABs, the choice of electrolyte is critical. Traditional liquid electrolytes present problems of flammability, leakage, and limited resilience under extreme conditions [7]. In contrast, solid or gel-like matrices solve these limitations. Polymer electrolytes offer design flexibility to achieve compact and lightweight battery configurations. In addition, their compatibility with various metals makes them adaptable for use in diverse metal–air battery systems [8]. However, their relatively lower ionic conductivity compared to liquid systems limits the power output of these batteries, requiring the incorporation of organic/inorganic phases that provide the matrix with the ions capable of performing the conduction process [9]. Hence, the benefits of gel-type polymer electrolyte systems (GPEs) arise from their combination of the favorable attributes found in liquid-type electrolytes, like their high ionic conductivity, and solid-state electrolytes, diminishing the high interface resistance [10,11]. Hydrogel networks are typically stabilized through either covalent bonds or noncovalent interactions among the chains of macromolecules, a process known as crosslinking [12]. The hydrogels have the capacity to absorb and hold considerable quantities of water without compromising their integrity [13]. The formation of a gel-like structure occurs within the polymer when cross-linking takes place.

Biopolymers can be considered a great option to substitute the synthetic polymers because they provide a degree of functionality [14] and abundance that is often lacking in the majority of reported synthetic polymers. Starch (A) is one of the most prevalent biopolymers in nature. Because of its affordability, renewable nature, availability in nature, and biodegradability, several scientists have shown an interest in developing commercial applications using starch and its derivatives [15]. However, the drawbacks of starch include fragility, excessive hydrophilicity, and poor mechanical and stability qualities. Chitosan (CH), the second most abundant polymer after cellulose, is more widely used to produce hydrogels as it stands out as the most valuable functional material for various applications in the field of polymer electrolytes due to its exceptional properties, including biocompatibility, biodegradability, non-toxicity, and absorption behavior [16]. Because of its characteristics of being an alkaline polyelectrolyte, chitosan can retain its chemical and thermal stability up to 200 °C while maintaining a satisfactory mechanical strength. Furthermore, the hydroxyl and amino groups present in the chitosan backbone enhance its hydrophilicity within the cells, which would be advantageous for the operation of GPEs [17].

CH:A hydrogels can be synthesized by either physical or chemical crosslinking methods. In our previous work, the chemical crosslinking using epichlorohydrin (EPI) was achieved. Physical crosslinking can be employed to strengthen the crosslink network. Physical hydrogels can be produced through a process involving repeated freeze–thaw (F-T) cycles. This process entails using concentrated aqueous solutions containing polymers that can form weak physical crosslinks. Freeze-drying using lyophilization is another low-cost physical crosslinking technique that creates uniform porous polymeric membranes with little shrinkage and great mechanical strength through the sublimation process at low temperatures and under vacuum [18]. The final properties of the hydrogels may vary depending on the freezing circumstances employed in the preparation such as temperature, time, and number of cycles [19]. In this study, chitosan–starch–epichlorohydrin electrolytes were synthesized with F-T cycles followed by freeze-drying techniques. The membranes were immersed in 12 M KOH solution. The effects of the crosslinking methods and different ratios of starch content on the structure, morphology, and thermal, mechanical, and electrochemical properties were assessed. Finally, primary battery tests were performed to assess the bulk resistance, power density, and specific capacity of the cell prototypes of this study.

## 2. Materials and Methods

Food-grade chitosan, 90.6% deacetylated [20] (purity: 100%, BioFitnest, Lindon, UT, USA), was used. Avocado seed starch was produced in the laboratories of Yachay Tech University according to a previously reported protocol [21]. Analytical-grade EPI, anhydrous glacial acetic acid (purity: 100%; density: 1.05 kg/L), and KOH pellets (purity ≥ 85.00%) were acquired from Sigma Aldrich (St. Louis, MO, USA). Distilled water was chosen as the solvent for all the solutions. For the electrochemical characterization, Pt plates (99.97%) and Zn discs (99.99%) were purchased from Goodfellow (Hamburg, Germany). For the battery tests, SIGRACET^®^ 39 B slides (10 mm width, 15 mm length, 0.4 mm thick) impregnated with a 1 mg·cm^−2^ catalyst mass loading of commercial catalytic ink along with Pt/C (20% wt) were employed as the cathode. High-purity Zn foil (10 mm width, 15 mm length, 0.2 mm thick) (purity: 99.99%, Yunexpress Inc., Shenzhen, China) was employed as the anode.

### 2.1. Synthesis of the CH:EPI and CH:A Hydrogels

**Figure 1:**
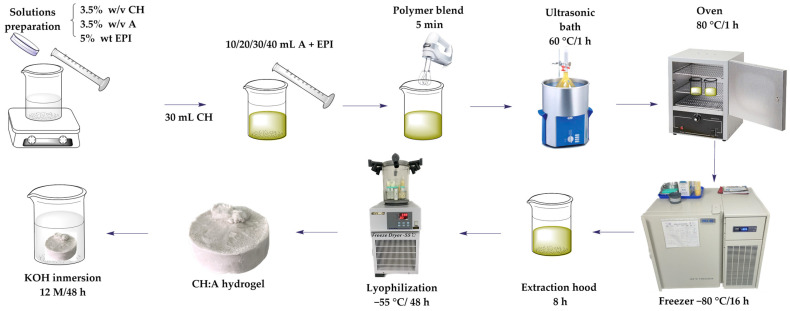
Synthesis of chitosan–avocado starch hydrogels using physical crosslinking techniques.

The synthesis of the hydrogels followed a similar procedure to the one previously reported by our research group [22], with some modifications. Briefly, homogenous solutions of 3.5% wt. of starch and 3.5% wt. of CH (in 1% wt. of acetic acid) were prepared separately. For starch dissolution, the mixture was heated at 80 °C. A fixed amount of CH (30 mL) was mixed with varying amounts of starch as indicated in Table 1. Then, 5% wt. of EPI was added to the mixture according to the polymers’ ratio and homogenized with an immersion blender. The resulting samples were placed in an ultrasound bath for 1 h at 60 °C and 40 kHz, followed by an additional 1 h drying step in an oven at 80 °C. The freezing strategies were implemented, involving a series of five freeze-thawing cycles (−80 °C for 16 h, followed by room temperature for 8 h). Subsequently, the resulting membranes were freeze-dried at −55 °C and 76 mTorr, using a freeze-dryer (Operon, Gimpo, Korea) for a duration of 48 h. The schematic representation of the hydrogel synthesis process is provided in Figure 1. Additionally, a starch-free hydrogel was synthesized to assess the effects and variances in comparison to the hydrogels proposed in this study. Following the synthesis, the obtained membranes were stored in a desiccator prior to the characterization procedures. The hydrogels intended for electrolyte testing were immersed in a 12 M potassium hydroxide solution for a duration of 48 h for characterization purposes. To distinguish this particular set of membranes, the identifier “Sw” was added to the hydrogel codes (Table 1).

### 2.2. Structural Morphology Characterization

Fourier transformed-infrared spectroscopy (FTIR) was conducted in the solid state with attenuated total reflectance (ATR) to analyze the functional group changes in the polymer matrices with the spectra obtained using a Cary 630 spectrophotometer (Agilent Technologies Inc., Santa Clara, CA, USA) equipped with a 1-bounce Diamond ATR accessory. Spectra were recorded in the range of 4000–400 cm^−1^, with 64 scans and a resolution of 4 cm^−1^. The crystallinity of the samples was evaluated at RT with the X-ray diffraction (XRD) patterns. X-ray diffractograms were obtained in a computer-controlled Rigaku Mini-flex-600 (Rigaku, Tokyo, Japan). The measurements were performed with a D/tex Ultra 2 detector 26. The X-Ray generator was placed in a sealed tube with a Ni-filtered Cu Kα radiation source (15 mA, 40 kV, λ = 0.15418 nm). The chosen angular region was 2θ = 5°–80°, with a step width of 0.01°. Match! Software version 2 [23] (Crystal Impact, Bonn, Germany) was employed to quantify the degree of crystallinity (DOC) of the samples. Surface micrographs were obtained using scanning electron microscopy (SEM) with a JEOL JSM6010/LV microscope (JEOL Ltd., Tokyo, Japan). Elemental analysis was performed on the swollen samples and on the samples after they were used in the battery prototypes with energy-dispersive X-ray spectroscopy (EDX) using an EDX TEAM analysis system integrated into the SEM equipment. Thermal properties of the materials were evaluated using thermogravimetric analysis (TGA) with a DSC-TGA Q600 (TA Instruments, New Castle, DE, USA). The chosen range was defined between RT to 800 °C with a heating ramp of 10 °C/min under N_2_ atmosphere. Swelling behavior of the electrolytes was studied by recording the weight of the samples before and after 48 h of immersion in 12 M KOH solution The swelling ratio (SR) was obtained with Equation (1):

(1) $$SR=\frac{W_{T}-W_{0}}{W_{0}}$$

where W_T_ and W_0_ are the swollen and initial membrane weights, respectively.

The biodegradability of the hydrogels in soil was estimated using commercial soil used for crops, following a method similar to the one reported by Michelle et al. [24]. Membranes of ∼0.5 cm thickness and 1.0 cm^2^ in area were buried in the soil at RT in triplicate. Soil was watered every 3 days. Samples weights were recorded at several time intervals after vacuum drying for 24 h.

### 2.3. Electrochemical Studies

The electrochemical behavior of the hydrogels was evaluated using electrochemical impedance spectroscopy (EIS) in a VIONIC instrument (Metrohm, Quito, Ecuador). The frequency range for the EIS was set from 100 kHz to 1 Hz. The proposed cell configuration was conformed of two 1 cm^2^ Pt blocking electrodes with the sample in between. This test was performed at different temperatures in the range of 0 °C to 60 °C (±1 °C precision, after 5 min of stabilization) with the aid of a Julabo circulator (Polyscience, −40 °C, 15 L, Niles, IL, USA). Four impedance measurements were carried out for each hydrogel. The ionic conductivity (σ) was determined with the use of Equation (2):

(2) $$\sigma =\frac{l}{A\times R_{b}}$$

where A is the area of the Pt electrode, l is the thickness of the hydrogel, and R_b_ is the bulk resistance obtained from the *X*-axis intersection of the impedance curve.

The activation energy (Ea) for each swollen sample was determined using the Arrhenius Equation (3) with a linear fit defined as the relationship between the logarithm of ionic conductivity and 1000/temperature:

(3) $$\sigma =\sigma_{0}exp(-\frac{Ea}{K_{b}\times T})$$

where T is the absolute temperature, K_b_ is the Boltzmann’s constant, and σ_0_ is a pre-exponential factor [25]. Data processing was performed in the OriginPro 2016 software (version 9) [26].

Cyclic voltammograms (CV) were performed in a symmetrical two-electrode Zn/hydrogel/Zn cell with 0.5 cm^2^ non-blocking Zn discs, at a sweep rate of 50 mV·s^−1^ in a symmetrical potential window from −1.5 V to +1.5 V.

Battery prototype tests were conducted with a previously reported configuration [27], without the use of the reservoir, placing the hydrogels between the Zn-Pt/C electrodes. The tests were performed in an AMETEK^®^ VersaSTAT 3 potentiostat/galvanostat (Princeton Applied Research, Berwyn, IL, USA). The cathode was designed with SIGRACET^®^ 39 B slides (15 mm length × 10 mm width, 0.4 mm in depth) impregnated with commercial catalytic ink (1 mg·cm^−2^) and Pt/C (20% wt.). The anode was designed to be a piece of polished high-purity Zn foil (15 mm length × 10 mm width, 0.2 mm in depth), (purity 99.9%, Yunexpress, Shenzhen, China). EIS spectra were evaluated at the OCP in a frequency range of 100 kHz to 0.1 Hz. The polarization curves were performed with a discharge current density of 20 mV·s^−1^, and the cut-off voltage was 0.2 V. The battery was discharged along different current densities for 300 s each. The specific capacity was determined in a constant current density of 3 mA·cm^−2^, along with the zinc mass loss from the test, and the equation for this is stated elsewhere [28].

## 3. Results

### 3.1. X-ray Diffraction Patterns

**Figure 2:**
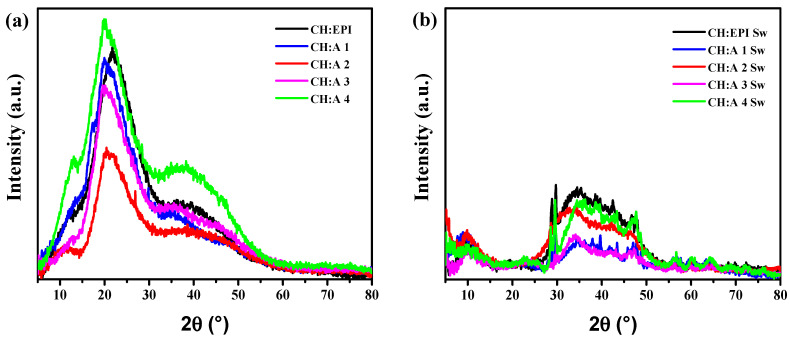
XRD patterns of CH:EPI and CH:A hydrogels with different amounts of starch: (**a**) before and (**b**) after hydration in a 12 M KOH solution.

In the XRD patterns obtained for the dried hydrogels (Figure 2a), the peak with the highest intensity is identified at 2θ = 21.8° for the CH:EPI membrane. This peak is related to the amine II (−NH_2_) of the chitosan structure [29]. However, in the presence of starch, this peak undergoes a shift to 2θ = 20.4°–20.1° and exhibits broadening as the starch content increases. This broadening is due to the destruction of the original crystalline structure of CH due to the intermolecular interaction between the CH and A chains. Similar behavior has been observed by other authors [30]. Since the shift moves towards lower 2θ values, it indicates an increase in interchain spacing. The dried samples present a broad peak of lower intensity than the previously discussed peak, with a center at 2θ = 39.7°. This peak is reported for chitosan when combined with other polymers such as PVA [31] and carboxymethylcellulose [32]. Furthermore, a peak at 2θ = 12.7° is identified for CH:A 4 hydrogel, which corresponds to the (111) plane of starch [33]. This peak is noticeable at a lower intensity for the other CH:A samples.

In the case of the swollen samples (Figure 2b), the insertion of potassium hydroxide in the polymer matrix is corroborated by the destruction of peaks observed in the XRD patterns of the dried hydrogels. Moreover, these peaks exhibit a marked reduction in intensity. These observations suggests that the crystallinity decrease is caused by the insertion of ions into the polymer chains, forming complexes and rearranging the hydrogen bonding [34]. For these hydrogels, the crystallinity decrease is evidenced by the calculated DOC (Table 2). The CH:A 3 Sw sample showed the lowest Xc value of 8.9%, which is relevant for the study since the crystallinity degree is closely linked to the swelling behavior of this type of materials [35]. Additionally, ions exhibit an improved mobility within amorphous phases, thereby enhancing the ionic conductivity of the resultant electrolyte [36].

### 3.2. ATR-FTIR Spectra

**Figure 3:**
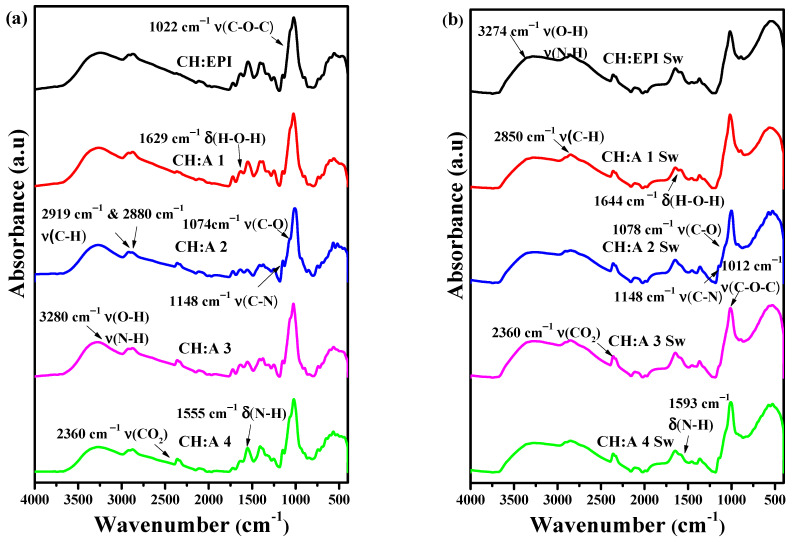
ATR-FTIR spectra of CH:EPI and CH:A hydrogels with different amounts of starch: (**a**) before and (**b**) after hydration in a 12 M KOH solution.

In our previous study, we reported the characteristic bands of chitosan and starch observed in the spectra, in addition to elucidating the reaction mechanisms associated with the incorporation of EPI into these two biopolymers [22]. In the FTIR spectra of dry membranes (Figure 3a), characteristic peaks typical for starch and chitosan were identified in the range of 3500 to 3000 cm^−1^, corresponding to the O-H and N-H stretching vibrations of the pyranose ring. Furthermore, to confirm the presence of these functional groups, the C-O and C-N stretching vibrations were observed at 1074 and 1148 cm^−1^ [37]. The peaks at 2919 cm^−1^ and 2880 cm^−1^ and 1022 cm^−1^ can be assigned to the C-H_2_ asymmetric stretching and C-O-C stretching vibrations. In addition, the O-H and N-H bending bands were observed at 1629 cm^−1^ and 1555 cm^−1^. Regarding the swollen hydrogels (Figure 3b), the main differences in the spectra compared to the dry hydrogels can be found in shifts and band intensities. The O-H and C-N stretching bands in the region of 3500 to 3000 cm^−1^ exhibited broadening and reduced sharpness and also shifted to 3274 cm^−1^. This is attributed to the immersion of hydrogels in water, forming a higher number of hydrogen bridges between their polymeric matrices [38]. Bands associated with the C-O-C and C-H vibrational motions were located at 1012 and 2850 cm^−1^. The already mentioned peaks at 1644 cm^−1^ and 1593 cm^−1^ correspond to O-H and N-H bending vibrations.

Another notable change that is observed is the reduction in the band intensity of the primary amine with a shift to a higher wavenumber of 1593 cm^−1^. As elucidated by R. Kasaai, this shift is likely attributable to the induction of a more amorphous state, which can be attributed to the presence of hydroxyl groups [39]. It can be inferred that the amino groups of chitosan have contributed to this shift. Compared with its counterpart, the membranes synthesized using the casting method [22], there is a noticeable difference in the intensity of the C-O-C stretch bond vibrations when immersed in the ionic solution due to the freeze–thaw techniques proposed by the synthesis method [40,41].

In addition, before and after immersing the lyophilized membranes in 12 M KOH, a peak is observed near 2300 cm^−1^ pointing to a possible interaction of the solution with the CO_2_ of the medium. Iles et al. [38] mention that KOH is used to capture carbon dioxide from the medium producing K_2_CO_3_ [42]. However, despite the fact that the membranes were synthesized in the presence of CO_2_ without a controlled atmosphere, the presence of potassium salts such as K_2_CO_3_ was not observed around 1350 cm^−1^, in contrast to the membranes synthesized by the casting method. This represents a great advantage when tested in zinc–air batteries since the deposit of this carbonate on the air electrode would be avoided, preventing the blockage of the oxygen transfer and increasing the performance of the battery [43].

### 3.3. Biodegradability Studies

The biodegradability of the materials was studied by burying the hydrogels in soil for 58 days. The degradation process of samples was evident over time, marked by an increment in their fragility and susceptibility to breakage. The weight retention as a function of the buried time was evaluated (Figure S1a). The materials synthesized with starch are capable of degrading to at least 40% of their initial weight after 2 months of being buried. The hydrogels are not only capable of swelling but also susceptible to moisture loss, depending on the environmental conditions. These characteristics represent a significant limitation when contemplating their use in ZABs [44]. The weight loss of the hydrogels soaked in 12 M KOH was studied over a period of 85 days (Figure S1b). The membranes underwent rapid dehydration in the first five days with weight losses of 30–20%. After this period, a weight gain can be observed and can be attributed to the absorption of moisture from the medium due to the hygroscopic nature of the hydrogels [45]. Beyond 20 days, the rate of water loss gradually diminished. Finally, after 30 days of study, the electrolytes reached a steady state with a remanent weight percentage of 60–50% wt.

### 3.4. Surface and Elemental Analysis

**Figure 4:**
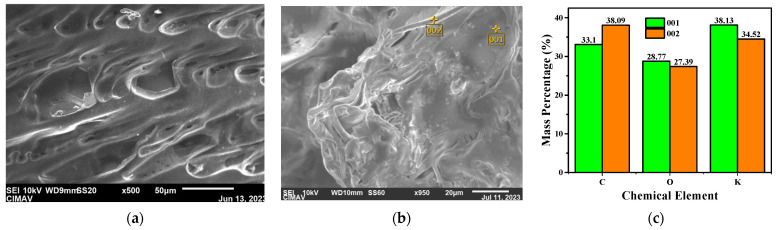
SEM micrographs of the CH:A 1 membrane in the (**a**) dried state, 950× magnification, and (**b**) in the swollen state, 250× magnification, and (**c**) the elemental composition of the CH:A 1 Sw electrolyte performed at (**b**) 001 and 002.

SEM micrographs of the CH:A 1 hydrogel (Figure 4a) exhibited surface pores with a lamellar structure, as a consequence of the freezing–thawing cycles. Moreover, no granules were observed, suggesting the complete complexation of the components of the hydrogel, as previously reported [46]. When compared to its casting method counterpart [22], the physical crosslinking generated denser pores, related to the higher KOH absorption capability of these hydrogels, necessary for the conduction mechanism in the material. The surface was analyzed after the absorption of the ionic solution (Figure 4b), revealing the generation of some granules. Nevertheless, these appeared in much smaller quantities compared to the ones in the casting membranes. The granules and the overall surface were analyzed using EDS, and the mass composition was obtained (Figure 4c). In the case of the evaluated granule (zone 001), potassium salts were identified, but no chlorine traces from EPI were found. Furthermore, KCl might not exist in these materials, contrary to the one formed in the casting-dried hydrogels [22]. Furthermore, the similar radii of carbon, oxygen, and potassium in both zones suggests an adequate potassium fixation around the membrane.

After the discharge evaluation in the ZAB prototype, EDS mapping was performed in the CH:A 1 Sw hydrogel, where the element composition as mass was obtained for both the cathode- and anode-facing sides of the membrane (Figures S2 and S3). On the cathode-facing side, there was a deposition of zinc of approximately 8%. This observation constitutes a confirmation of the capacity of the electrolyte to enable the migration of ions from the electrode to the other side.

### 3.5. Thermogravimetric Analysis

**Figure 5:**
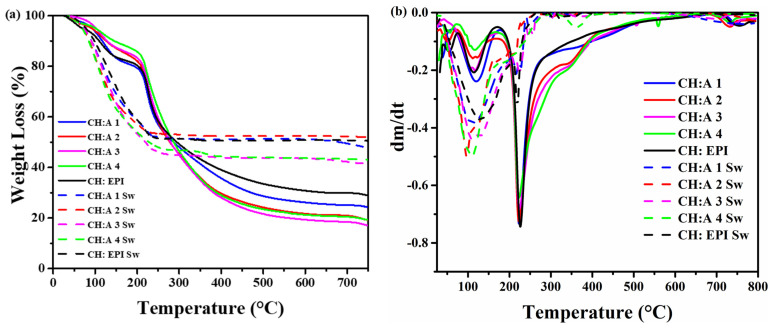
(**a**) TGA and (**b**) DTGA curves of modified hydrogels.

The design of GPEs that exhibit a satisfactory thermal stability is a crucial requirement to enhance the battery’s safety and minimize the risk of thermal runaway. TGA is a commonly employed method to evaluate the thermal stability of battery electrolytes. The thermogram for the membranes prior to their immersion in KOH solution reflects a first degradation region that deteriorates into two different processes as can be seen in the differential thermogram comprising the zones between 50 and 120 °C (Figure 5a). This initial mass loss of 6–11% wt. is attributed to water desorption. In addition, a second thermodegradation region can be observed in the region between 223–340 °C, corresponding to the pyrolysis of the biopolymers, the cleavage of glycosidic linkages, and the deacetylation of chitosan [47]. The total weight losses of CH:EPI, CH:A 1, CH:A 2, CH:A 3, and CH:A 4 hydrogels above 750 °C were found to be 27.56%, 23.34%, 18.33%, 16.39%, and 18.80%, respectively. The residual weight was probably ascribed to the residual carbon compounds by thermal decomposition.

After soaking the membranes in a 12 M KOH solution, two regions of decomposition were observed (Figure 5b). The first one is around 97 °C to 128 °C, which is attributed to the loss of water inside the polymeric matrices. The weight loss was around 29–35% wt., which is higher than that of the dry hydrogels due to the absorption of the ionic solution. In the second stage of mass loss at 216 to 220 °C, the loss of mass is attributed to the degradation of the polysaccharide structure of the electrolytes [48]. Finally, beyond 400 °C, the residue was 50.55, 51.21, 52.45, 43.73, and 43.92 for the CH:EPI, CH:A 1, CH:A 2, CH:A 3, and CH:A 4 electrolytes, respectively. Compared to the dry membranes, the inclusion of KOH in these membranes increases their thermal stability around 100 °C. In addition, their decomposition rate is lower than that of dry membranes, which can be attributed to the KOH solution and a high chemical crosslinking present even at high temperatures.

### 3.6. Impedance Studies

**Figure 6:**
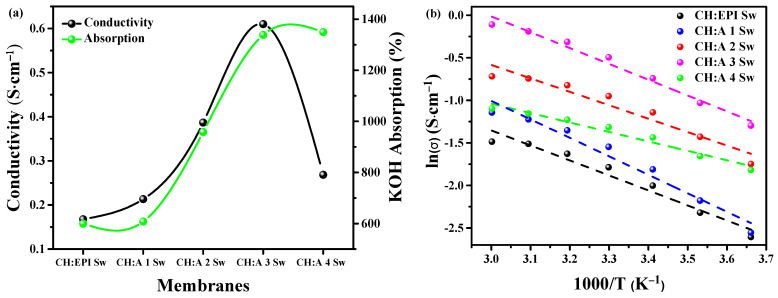
(**a**) Evaluation of the ionic conductive values and the swelling ratios of the electrolytes. (**b**) Conductivity studies of the hydrogel samples at different temperatures.

The salt chosen to dop the polymer matrix plays an imperative role in the designed material, as it contributes the ionic species that enhance the ionic conductivity of the material. Hence, the swelling degree is evaluated in relation to the ionic conductivity (Figure 6a). The Nyquist diagrams corresponding to the EIS studies at 30 °C are presented in Figure S4. Figure 6a shows a clear increase in the absorption of KOH as more starch polymer is present in the membrane, with 607% of SR for the starch-free hydrogel and up to 1338% for the CH:A 3 Sw sample. This tendency is lost in the CH:A 4 Sw membrane, as it is the one with lesser amount of chitosan than starch. This is comparable to the observed conductivity values, with a maximum of 0.61 S·cm^−1^ for the CH:A 3 Sw electrolyte, falling down to 0.27 S·cm^−1^ for the CH:A 4 Sw membrane. As first proposed in the study on casting-dried membranes, starch is incorporated into the proposed synthesis to improve the integrity and resistance of the chitosan-only hydrogel. The CH:A 4 Sw membrane is also proposed in this study, with which it is found that a hydrogel with a higher amount of starch than chitosan causes a decrease in the desired electrochemical properties. In addition, the superior characteristics of the hydrogels presented in this study are shown with the less swollen hydrogel that achieves an SR of 607%, while for the best casting-dried hydrogel, a maximum swelling degree of 94.5% was reported. In terms of ionic conductivity, hydrogels of this study surpassed the casting hydrogels from our previous study [22], which exhibited a conductivity of 0.027 S·cm^−1^, and this value was exceeded by more than 20 times by the best sample of this study. The enhancement in the properties is explained in terms of the obtained morphology by the physical crosslinking strategies that provide a system capable of holding more ionic solution and paths for the ions to move more easily in the system [49].

The relationship between the ionic conductivity and temperature was evaluated to determine the conductive mechanism model that best fits the materials (Figure 6b). A linear dependence was obtained between the plot of ln (σ) versus 1000/T for each curve. This mathematical model is attributed to Arrhenius theory, which states that the ionic conductivity is thermally assisted [50]. The hydrogels held these ionic conductivity behavior up to 60 °C, an upgrade in contrast to the casting-dried membranes that only maintained this behavior up to 50 °C. This points to an enhanced stability in the conduction process for this set of hydrogels. From the proven conductive mechanism, the activation energy (Ea) can be calculated with Equation (3). An average Ea of 0.12 eV was obtained (Table 3), as a representation of the energy requirement for the ion to move from one site to another in the system [51].

### 3.7. Cyclic Voltammetry

**Figure 7:**
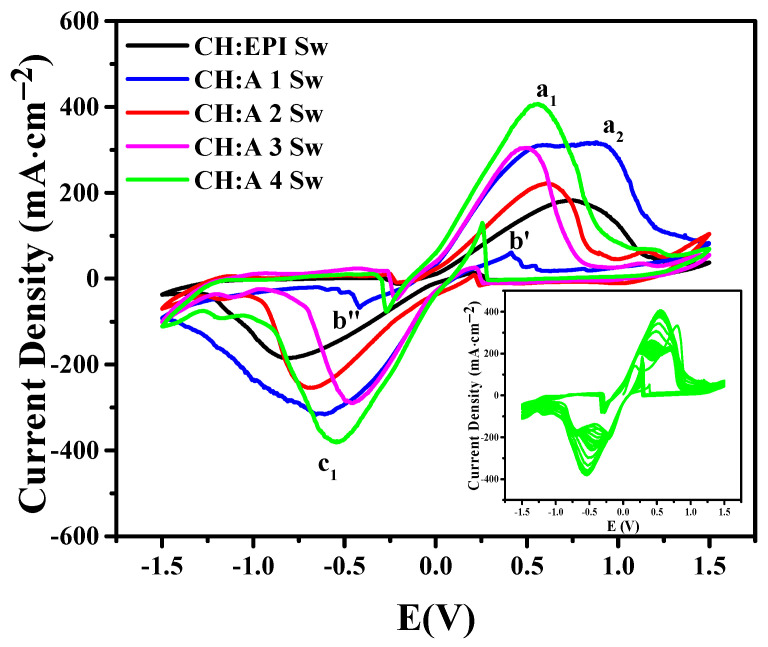
Cyclic voltammperograms of CH:EPI Sw and CH:A Sw hydrogels with different starch contents. Inset: 30 consecutives cycles of the CH:A 4 Sw hydrogel.

Cyclic voltammetry (CV) studies were carried out to confirm the ionic transport into the membranes and the electrochemical stability of the electrolytes. The anodic and cathodic peaks are observed at 0.56 V and 0.55 V, respectively (Figure 7), as a consequence of the oxidation and reduction processes. The anodic peaks a_1_ and a_2_ correspond to the oxidation of Zn (0) to Zn (II) with the formation of zincate species, Zn(OH)_4_^2−^ and Zn(OH)_3_^−^, respectively. The second species is created due to the depletion of the hydroxyl groups present in the solution in the vicinity of the electrode surface, after which a pre-passive film is formed at potentials more positive than the a_1_ peak. The anodic peak (b′) and cathodic peak (b″) observed when performing the cathodic and anodic scanning, respectively, scanning are related to the oxidation processes of zinc after the pre-passive layer, which is formed on the electrode surface, is dissolved during this process. According to Ming Cai et al., the composition of the pre-passive layer could be made up of intermediate species before a well-defined ZnO is formed [52]. The c_1_ cathodic peak is attributed to the reduction of Zn^2+^ to Zn^0^. In addition, an increase in the intensity of the peaks can be inferred by the increasing proportion of starch associated with a large amount of water absorption and ionic solution. The rise in current density can be attributed to the absorption of water and KOH during the soaking process. A maximum current density for CH:A 4 of 400 mA∙cm^−2^ was reached for a total of 30 consecutive cycles (inset), affirming a highly steady performance in cycling [53]. In addition, for each of the hydrogels with their different compositions, a quasi-reversible behavior was observed. In conclusion, these voltammograms demonstrate that Zn^2+^ ions exhibit mobility within the GPEs [54], and Zn can undergo both dissolution into and deposition from the membranes. This property is vital for their potential utilization in zinc batteries [55].

### 3.8. Battery Evaluation

**Figure 8:**
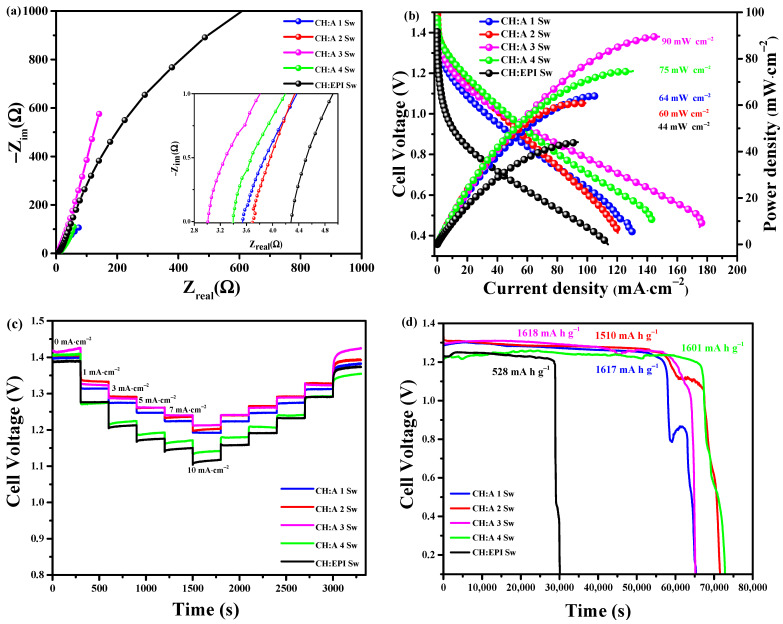
(**a**) PEIS studies; (**b**) power densities and discharge polarization curves; (**c**) discharge tests at different demanded currents; and (**d**) discharge curve for the ZABs at 3 mA cm^−2^.

The OCP varied between 1.39 V and 1.48 V. From the Nyquist plot, bulk resistances values between 3 Ω and 4.3 Ω were obtained, and these values being below 10 Ω suggest that there is good contact between the electrolyte and the electrodes. The performance of the CH:A Sw electrolyte in electrochemical terms surpasses that of the CH:EPI Sw electrolyte (Figure 8a) and is also superior to other polymer electrolytes, such as PAM6M with a bulk resistance of 6.79 Ω [56] and PVA-gelled electrolyte (0° bending angle) with a bulk resistance of approximately ~4 Ω [57]. The discharge polarization curves and their power densities are displayed in Figure 8b. From the polarization curves, it is evident that in the activation region, the zinc–air battery with different hydrogels exhibited a voltage between 1.38 and 1.48 V. Membranes with starch exhibited a similar region in terms of ohmic losses compared to the starch-free hydrogel, which displayed a higher ohmic resistance, resulting in a greater voltage drop and a decline in battery performance. From this graph, it is also noticeable that, as the starch proportion is varied, there is a difference in the mass transport region. Furthermore, the current density increases from 113 to 143 mA for the CH:EPI Sw to CH:A 4 Sw hydrogels, reaching a maximum of 175 mA∙cm^−2^ for the CH:A 3 Sw hydrogel. From the power density curves, a higher performance can be observed for the CH:A 3 Sw hydrogel, reaching a maximum of 90 mW∙cm^−2^. This value is higher compared to those reported in the literature for similar systems such as Starch–Gel–KOH with a maximum value of 84 mW cm^−2^ [58], Fiber–KOH with 68.7 mW∙cm^−2^ [59], PVA–KOH with 71 mW∙cm^−2^ [60], and its counterpart CH:A 3 Sw using the casting method with 8.82 mW cm^−2^. A test demanding different current densities was performed to determine the voltage requirements and recovery capacity of the prototype with the proposed hydrogels (Figure 8c). The CH:A 3 Sw hydrogel required 0.20 V to achieve a demand of 10 mA·cm^−2^. In the case of the CH:A 4 Sw and CH:EPI Sw hydrogels, this value increased to 0.25 V and 0.28 V, respectively. Hence, a voltage difference of 50 mV to 80 mV is observed between the hydrogels. In the reverse demand of different current densities, the tested batteries required almost the same voltage as in the beginning of 3 mA·cm^−2^. The difference in voltage was lower than 0.01 V, an indicator of the appropriate recovery of the batteries used in the test. Overall, the CH:A 3 Sw hydrogel required the lowest overpotential to deliver any of the fixed currents. To investigate the discharge performance of the ZAB prototypes with the hydrogels, the galvanostatic discharge test was performed at a current density of 3 mA·cm^−2^ at RT (Figure 8d). The CH:EPI Sw electrolyte-based ZAB had a discharge voltage of 1.24 V, with an operation time of approximately 8 h. The best performance was achieved by the CH:A 3 Sw electrolyte-based ZAB, which showed a stable discharge voltage of 1.29 V and an operation time of 18 h. Based on the weight loss of Zn anode, the specific capacity of this ZAB was calculated to reach up to 1618 mAh·g^−1^. An improvement is observed in comparison to the values reported in the literature for other zinc-ion batteries and for zinc–air batteries, the PANa electrolyte (PANa containing Zn(CH_3_COO)_2_ (0.2 M) and KOH (6 M)) demonstrated capacities of ∼260 mAh g^−1^ (NiCo hydroxide) and ∼800 mAh g^−1^ (Zn), respectively [61]. These results point to the competitive voltage efficiency of the proposed material.

## 4. Conclusions

The innovative synthesis approach employed a combination of chemical and physical techniques, yielding materials tailored for a potential application in ZABs. This synthesis induced noticeable shifts in the frequencies and intensities of signals within the ATR-FTIR spectra, providing clear evidence of the successful completion of the crosslinking process. XRD patterns indicated a reduction in material crystallinity, a critical factor influencing swelling ratios and conduction pathways within the hydrogels. Insightful analysis of SEM micrographs revealed the porous lamellar structure of the dried membranes, while the swollen electrolytes exhibited the proper incorporation of ions into the polymer matrix. Furthermore, thermograms confirmed the enhanced thermal stability of the membranes upon the incorporation of the ionic solution. The hydrogels were able to absorb 14 times their own weight of KOH 12 M solution. Of particular significance is that the CH:A 3 Sw membrane displayed an exceptional electrochemical performance, boasting a maximum ionic conductivity of 0.61 S·cm^−1^ and a remarkable Arrhenius conduction mechanism extending up to 60 °C. Cyclic voltammetry analysis demonstrated a quasi-reversible response in the GPEs during the oxidation and reduction stages, showing a peak intensity of approximately 400 mA·cm^−2^. Finally, the prototype testing of the ZAB prototype exhibited a discharge value of 1618 mA h·g^−1^ and a power density of 90 mW·cm^−2^. These results emphasize the environmentally friendly and sustainable potential of these hydrogels as electrolytic solutions for application in zinc–air batteries (ZABs) and other metal–air battery systems.

## Figures and Tables

**Table 1:** Codes based on names and compositions of the GPEs used in this study.

Hydrogel	Electrolyte
CH:EPI	chitosan:epichlorohydrin
CH:A 1	chitosan:starch EPI (3:1)
CH:A 2	chitosan:starch EPI (3:2)
CH:A 3	chitosan:starch EPI (3:3)
CH:A 4	chitosan:starch EPI (3:4)
CH:EPI Sw	chitosan:epichlorohydrin EPI in 12 M KOH
CH:A 1 Sw	chitosan:starch (3:1) EPI in 12 M KOH
CH:A 2 Sw	chitosan:starch (3:2) EPI in 12 M KOH
CH:A 3 Sw	chitosan:starch (3:3) EPI in 12 M KOH
CH:A 4 Sw	chitosan:starch (3:4) EPI in 12 M KOH

**Table 2:** Degree of crystallinity (DOC) estimated from the XRD diffractograms.

Hydrogel	DOC (%)
CH:EPI	14.6
CH:A 1	15.5
CH:A 2	13.4
CH:A 3	14.8
CH:A 4	16.7
CH:EPI Sw	12.6
CH:A 1 Sw	11.3
CH:A 2 Sw	11.3
CH:A 3 Sw	8.9
CH:A 4 Sw	13.4

**Table 3:** Electrochemical and battery values for the electrolytes.

Electrolyte	σ (S·cm^−1^)	Ea (eV)	ΔEp (V)	Bulk Resistance (Ω)	Specific Capacitance (mAh g^−1^)	Power Density (mW cm^−2^)
CH:EPI Sw	0.17	0.15	0.05	4.29	528	44
CH:A 1 Sw	0.21	0.19	0.09	3.55	1617	64
CH:A 2 Sw	0.39	0.14	0.07	3.71	1510	60
CH:A 3 Sw	0.61	0.16	0.04	3.02	1618	90
CH:A 4 Sw	0.27	0.15	0.01	3.40	1601	75

## Data Availability

Data sharing is not applicable for this article.

## Supplementary Materials

The following supporting information can be downloaded at: https://www.mdpi.com/article/10.3390/polym15224398/s1, Figure S1: Weight retention results from the biodegradation of the dried samples (a) in composted soil and (b) at ambient temperature as a function of exposure time. Figure S2: Mapping of cathode-facing of CH:A 1 sw hydrogel. Figure S3: Mapping of anode-facing side of CH:A 1 sw hydrogel. Figure S4: Nyquist plot for impedance measurement of the CH:EPI Sw and CH:A Sw hydrogels.
