# Supplementary material for: Green Energy Storage: Chitosan-Avocado Starch Hydrogels for a Novel Generation of Zinc Battery Electrolytes
Source: Polymers (Basel). 2023 Nov 14;15(22):4398. doi: 10.3390/polym15224398 (PMC10675044; doi:10.3390/polym15224398)
Supplement: Supplementary file 1 [file polymers-15-04398-s001.zip › polymers-2699856-supplementary.pdf]

# Green Energy Storage: Chitosan-Avocado Starch Hydrogels for a Novel Generation of Zinc Battery Electrolytes

**María I. Cruz-Balaz [1], María Fernanda Bósquez-Cáceres [1], Anabel D. Delgado [2], Noé Arjona [3], Vivian Morera Córdova [1], Lorena Álvarez-Contreras [2,*] and Juan P. Tafur [1,4,*]**

[1] Grupo de Investigación Aplicada en Materiales y Procesos (GIAMP), School of Chemical Sciences & Engineering, Yachay Tech University, Urcuquí 100115, Ecuador; maria.cruzb@yachaytech.edu.ec (M.I.C.-B.); maria.bosquez@yachaytech.edu.ec (M.F.B.-C.); vmorera@yachaytech.edu.ec (V.M.C.)

[2] Centro de Investigación en Materiales Avanzados S.C. (CIMAV), Miguel de Cervantes No. 120, Complejo Industrial Chihuahua, Chihuahua 31136, Mexico; anabel.delacruz@cimav.edu.mx

[3] Centro de Investigación y Desarrollo Tecnológico en Electroquímica S. C., Pedro Escobedo, Querétaro C.P. 76703, Mexico; wvelazquez@cideteq.mx

[4] Departamento de Ingeniería Mecánica, Química y Diseño Industrial, Escuela Técnica Superior de Ingeniería y Diseño Industrial (ETSIDI), Universidad Politécnica de Madrid (UPM), Ronda de Valencia 3, 28012 Madrid, Spain

* Correspondence: lorena.alvarez@cimav.edu.mx (L.Á.-C.); jp.tafur@upm.es (J.P.T.)

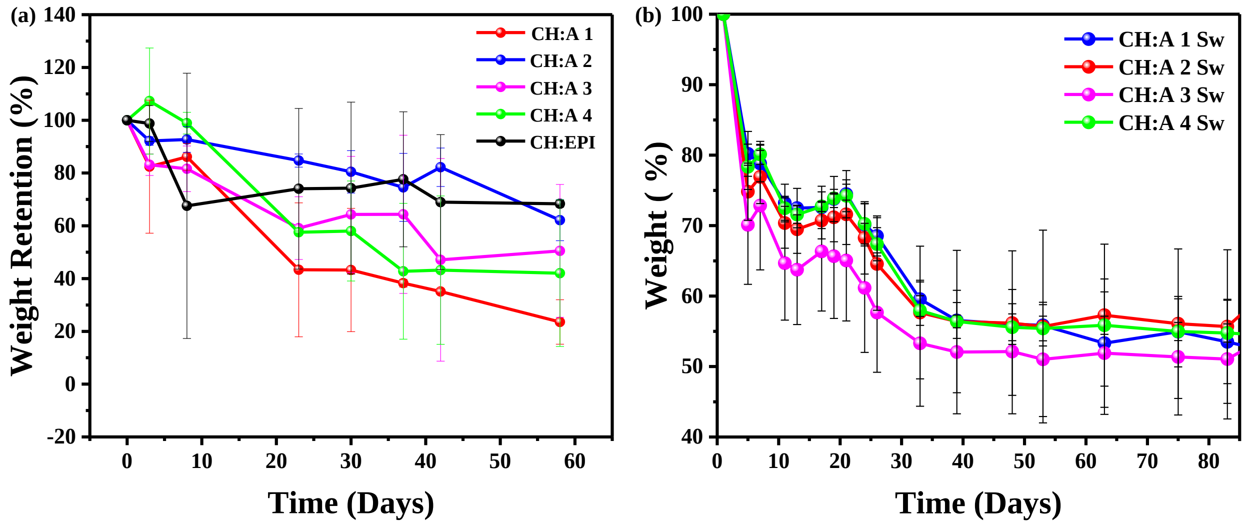


**Figure S1.** Weight retention results from biodegradation for the dried samples in (a) composted soil and (b) at ambient temperature as a function of exposure time.

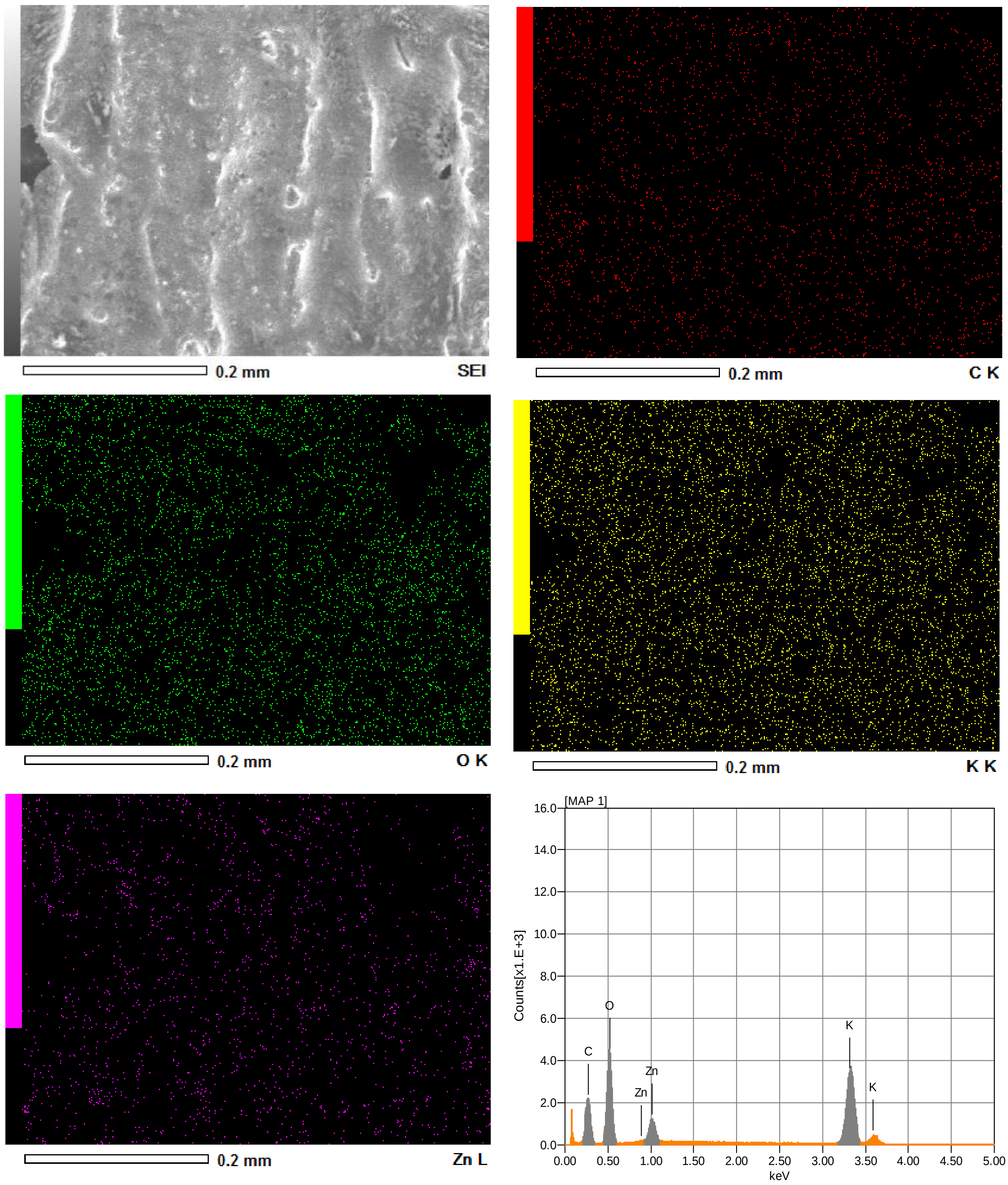


**Figure S2.** CH:A 1 sw cathode-facing side mapping.

| Chemical Formula | Mass% | Atom% | Sigma | Net | K ratio | Line |
|---|---|---|---|---|---|---|
| C | 13.60 | 25.01 | 0.02 | 50862 | 0.0031145 | K |
| O | 34.36 | 47.43 | 0.08 | 113275 | 0.0204018 | K |
| K | 43.98 | 24.84 | 0.16 | 179432 | 0.0864599 | K |
| Zn | 8.05 | 2.72 | 0.08 | 38578 | 0.0082465 | L |
| Total | 100.00 | 100.00 | | | | |

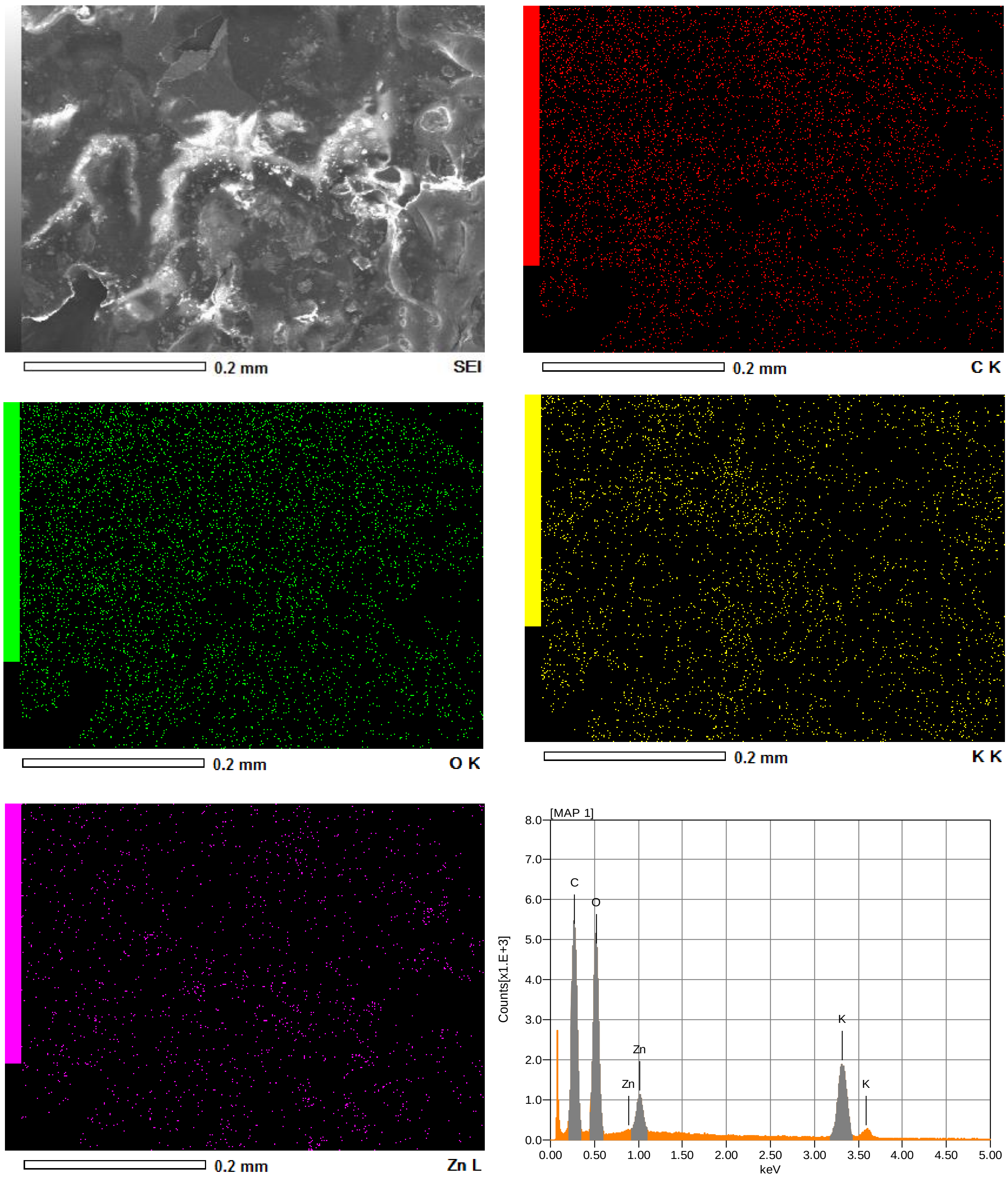


**Figure S3.** CH:A 1 sw anode-facing side mapping.

| Chemical Formula | Mass% | Atom% | Sigma | Net | K ratio | Line |
|---|---|---|---|---|---|---|
| C | 32.95 | 48.13 | 0.02 | 128315 | 0.0078574 | K |
| O | 35.56 | 38.99 | 0.08 | 125807 | 0.0226595 | K |
| K | 24.60 | 11.04 | 0.12 | 98315 | 0.0473745 | K |
| Zn | 6.89 | 1.85 | 0.07 | 33236 | 0.0071048 | L |
| Total | 100.00 | 100.00 | | | | |

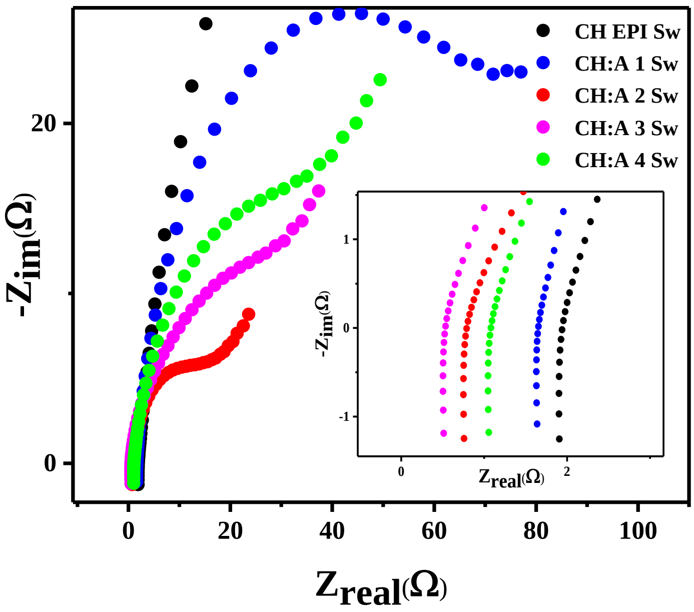


**Figure S4.** Nyquist plot for impedance measurement of the CH:EPI Sw and CH:A Sw hydrogels.
